# The Gut Microbiota and Short-Chain Fatty Acids Profile in Postural Orthostatic Tachycardia Syndrome

**DOI:** 10.3389/fphys.2022.879012

**Published:** 2022-06-02

**Authors:** Jeanne A. Ishimwe, Nicholas Breier, Mohammad Saleem, Paul D. Kastner, Annet Kirabo, Cyndya A. Shibao

**Affiliations:** Division of Clinical Pharmacology, Department of Medicine Vanderbilt University Medical Center and Department of Molecular Physiology and Biophysics, Vanderbilt University, Nashville, TN, United States

**Keywords:** POTS, gut microbiota, short-chain fatty acids, norepinephrine, hypotension

## Abstract

Postural orthostatic tachycardia syndrome (POTS) is a devastating chronic form of orthostatic intolerance associated with excessive heart rate increase without hypotension during upright posture. POTS patients exhibit increased circulating norepinephrine levels with exaggerated sympathetic nervous system response upon standing. Emerging evidence suggests a role for the gut microbiome in cardiovascular disorders. However, the etiology of POTS and whether the gut microbiome plays a role are not fully elucidated. We assessed whether the gut microbiome and fecal short-chain fatty acids were different in POTS patients (*N* = 25) compared to healthy control (*N* = 23) women. Patients underwent hemodynamic measurements while supine and upon standing. Fecal samples were collected and analyzed using shotgun sequencing and Liquid Chromatography-High Resolution Mass Spectrometry and dietary habits were measured with a fitness application. We found that POTS patients in the standing position had higher circulating norepinephrine and epinephrine levels and increased heart rate. There were no differences in diet composition between groups. Of note dietary salt intake was also similar despite the fact that these patients are advised to consume a high salt diet. Alpha and beta diversity were similar between groups. We observed no differences in bacteria at the phylum levels or Firmicutes to Bacteroidetes ratio. We found no significant differences at the genus level, but observed trends in certain bacteria. *Lachnoclostridium* genus were higher in POTS when compared to the control group. On the other hand, *Coprococcus* and *Coprobacter*, were lower in POTS patients compared to controls. Although our KEGG metabolic pathways indicated differences related to short-chain fatty acids (SCFAs), we found that both POTS patients and healthy controls had similar levels of SCFAs. These results suggest POTs per se may have limited effects on gut microbiota composition and derived SCFAs. Further studies are needed to assess the role of the alterations observed at the genus level.

## Introduction

Postural orthostatic tachycardia syndrome (POTS) is a chronic, disabling condition characterized by symptoms that occur on standing including lightheadedness, dizziness, and tachycardia without orthostatic hypotension. These symptoms are relieved, in part, by changing to a supine position (Low et al., 1995). The etiology is not fully understood, elevated circulating norepinephrine levels have been reported in POTS patients suggesting that increased sympathetic activity may play a role (Goldstein et al., 2002; Lambert et al., 2008). POTS affects 3 million Americans, with 80–85% of those affected being women especially of the child-bearing age (Zadourian et al., 2018).

The microbes in the gastrointestinal tract and their genetic material, most commonly known as the gut microbiome, play a role in modulating host health (Cryan et al., 2020; Elijovich et al., 2020). Gut microbiome homeostasis can be disrupted by factors such as diet, medication, and genetics. The resulting dysbiosis is associated with changes in gut wall permeability and changes in gut-derived metabolites have been implicated in pathological conditions (Kim et al., 2018; Ferguson et al., 2019; Ruan et al., 2020). Evidence suggests a role of the gut microbiome in regulating the autonomic nervous system (Zubcevic et al., 2019). Due to great variability in the microbiome across individuals, studies have largely focused on examining the association between gut dysbiosis and disease. Bacteria in the gut produce metabolites through fermentation of indigestible polysaccharides such as dietary fiber that mediate its effect on the host physiology. Short-chain fatty acids (SCFAs) like acetate, propionate, and butyrate are the main gut-derived metabolites and influence gut-brain communication by inducing the secretion of the gut hormones and neurotransmitters (Silva et al., 2020).

Gut dysbiosis is reported in neurological disorders such as depression and Parkinson’s (Chu et al., 2019; Tian et al., 2019; Nishiwaki et al., 2020). The role of the gut microbiome in POTS has not been investigated. The aims of this study were 1) to evaluate if POTS patients have dysbiosis of the gut microbiota compared with healthy controls and 2) to determine differences in SCFAs in stools in POTS patients and healthy controls.

## Methods

Study participants: We enrolled a total of 48 subjects, 25 had postural tachycardia syndrome, and 23 were healthy volunteers (Table 1). POTS patients were recruited from referrals to the Vanderbilt Autonomic Dysfunction Center. The study was approved by an institutional review board (Vanderbilt Human Research Protection Program) and all participants gave written informed consent. Further, the study was conducted in accordance with institutional guidelines and adhered to the principles of the Declaration of Helsinki and Title 45 of the US Code of Federal Regulations (Part 46, Protection of Human Subjects) and the study was registered in clinicaltrials.gov NCT03263819. Subjects were excluded if they reported the use of antibiotics for acute or chronic illness. Same household members were excluded as controls. Healthy controls were enrolled from the Nashville community, and they were excluded if they were a member of the household of an enrolled POTS patient.

**TABLE 1:** Demographic and clinical characteristics of the subjects.

	Control (*N* = 14)	POTS (*N* = 7)	*p* value (*t* test)
Sex	Female	Female	—
Race	White	White	—
Waist (cm)	78.4 ± 12.70	93.8 ± 16.23	0.0514
Hip (cm)	91.9 ± 12.0	110.5 ± 14.37	0.0114
Thigh (cm)	90.4 ± 14.02	84 ± 22.38	0.4956
Age (years)	31.1 ± 7.45	33.4 ± 5.48	0.4635
BMI	23.6 ± 4.20	27.9 ± 7.42	0.1042
Supine SBP (mmHg)	97.2 ± 8.82	102 ± 11.25	0.2946
Supine DBP (mmHg)	59.5 ± 7.95	62.6 ± 4.99	0.3655
Supine HR (bpm)	62.9 ± 10.63	77.1 ± 6.20	0.0041
Standing SBP (mmHg)	103.1±8.77	113.9 ± 15.76	0.0575
Standing DBP (mmHg)	71.1 ± 5.56	72.4 ± 9.07	0.6743
Standing HR (mmHg)	85.2 ± 13.42	115.9 ± 13.55	0.0001

Values are expressed as mean ± SD. SBP, systolic pressure; DBP, diastolic pressure; HR, heart rate; bpm, beats per minute.

Study Procedures: During the screening visit, the participants underwent a medical history review, physical examination, laboratory assessments (cell blood count, comprehensive metabolic panel, lipid profile), supine and standing plasma norepinephrine and autonomic function tests as described in a previous publication (Cheshire and Goldstein 2019).

Dietary data collection: Data on the food intake of participants were collected 2 weeks before the fecal sample collection. All enrolled subjects were asked to maintain their current diet for these 2 weeks during which they were asked to track their diet using the smartphone application MyFitnessPal during the 2 weeks before stool sample collection.

Fecal Sample Collection: Changes in medication and doses were not allowed during this run-up period. Fecal samples were collected at home by participants in 5 ml conical tubes containing media designed to stabilize RNA (OMNIgene Gut sample collection kit) for microbiome study and SCFAs analysis and brought to our laboratory for processing. The samples were stored at −80°C for further analysis of SCFAs.

Gut microbiota characterization: Samples’ DNA was extracted with PowerSoil Pro (Qiagen) automated for high throughput on the QiaCube HT (Qiagen), using Powerbead Pro Plates (Qiagen) with 0.5 and 0.1 mm ceramic beads. QC Samples were quantified with Quant-iT PicoGreen dsDNA Assay (Invitrogen). Libraries were prepared with a procedure adapted from the Illumina DNA Prep kit (Illumina). For BoosterShot^®^ (Shallow Sequencing, 2 M reads/sample), libraries were sequenced on an Illumina NextSeq using single-end 1 × 150 reads with a NextSeq 500/550 High Output v2 kit (Illumina). Sequences were trimmed to a maximum length of 100 bp before alignment and converted to a single fasta using shi7. DNA sequences were aligned to a curated database containing representative genomes in RefSeq for bacteria with additional manually curated strains (Venti). Alignments were made at 97% identity against all reference genomes. Every input sequence was compared to every reference sequence in Diversigen’s Venti database using fully gapped alignment with BURST. Ties were broken by minimizing the overall number of unique Operational Taxonomic Units (OTUs). For taxonomy assignment, each input sequence was assigned the lowest common ancestor that was consistent across at least 80% of all reference sequences tied for best hit. Samples with fewer than 10,000 sequences were also discarded. OTUs accounting for less than one-millionth of all species-level markers and those with less than 0.01% of their unique genome regions covered (and <1% of the whole genome) were discarded. The number of counts for each OTU was normalized to the average genome length. Samples with fewer than 10,000 sequences were discarded. Kyoto Encyclopedia of Genes and Genomes Orthology groups (KEGG KOs) were observed directly using alignment at 97% identity against a gene database derived from the strain database used above. The KO table and downstream tables contain the directly observed KO counts converted to relative abundance within a sample. The normalized and filtered data (low count filter: minimum count of 4; prevalence in samples by at least 20%; and low variance filter of 10% based on the interquartile range)were used for all downstream analyses using MicrobiomeAnalyst (Dhariwal et al., 2017; Chong et al., 2020).

Fecal short-chain fatty acid analysis: Fecal matter was weighed, diluted to a final density of 100 mg/ml in MeOH/H2O (2:1), and homogenized/extracted with a cordless Pellet Pestle tissue grinder equipped with disposable polypropylene mixers (Fisher). Insoluble debris was removed by centrifugation (12,000 × g, 30 min, 5°C); the supernatants were transferred to clean Eppendorf tubes and stored at −20°C until the day of analysis. SCFAs were measured by Liquid Chromatography-High Resolution Mass Spectrometry (LC-HRMS) in the bioanalytical core facility in the Vanderbilt Mass Spectrometry Research Center.

Statistical analysis: Alpha diversity by Shannon and Beta diversity by Bray-Curtis indices were calculated using MicrobiomeAnalyst by ANOVA and PERMANOVA, respectively. A random forest analysis and a zero-inflated Gaussian fit model (FDR<0.05) were used to determine features that are differentially abundant between groups (Roguet et al., 2018). Kyoto Encyclopedia of Genes and Genomes (KEGG) orthologs abundance levels were used to explore for overall functional profiling. The MicrobiomeAnalyst generated taxa abundances and SCFAs were further analyzed for timed differences using GraphPad Prism software (GraphPad Prism 7.01). Statistical analyses were performed by Student’s *t*-test for comparisons. Data are presented as means ± SE. A probability value < 0.05 was considered for statistical significance.

## Results

**FIGURE 1:**
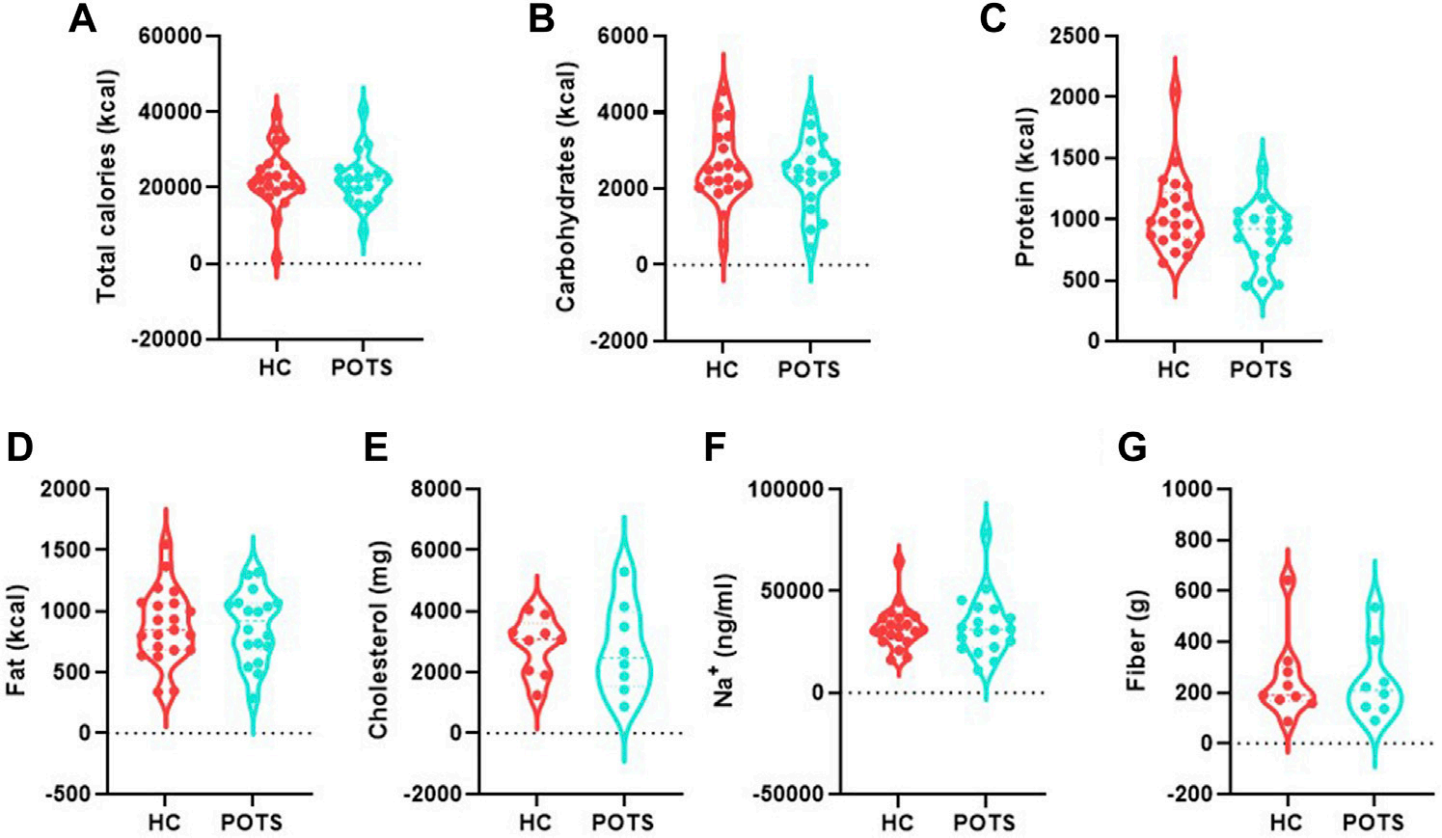
Dietary intake in POTS patients and healthy controls. Participants recorded dietary information for 2 weeks using the MyFitnessPal application. **(A)**Total calories; **(B)**carbohydrates; **(C)** protein; fat **(D)**; **(E)** cholesterol; **(F)** sodium; and **(G)** fiber. Data are presented as mean ± SEM. Statistical analyses were performed using Student’s *t*-test. *N* = 25 for POTS and *N* = 23 for healthy control participants and *p* < 0.05 was set for statistical significance.

Dietary Data: We obtained dietary information from the information study participants logged in the MyFitnessPal application. Caloric intake was similar between groups (Figure 1A). Dietary composition is a major modulator of the gut microbiota. Hence, we determined if there were any differences in the specific food components. We found that levels of carbohydrates (Figure 1B), protein (Figure 1C), total fats (Figure 1D), and cholesterol (Figure 1E) were similar between POTS patients and healthy controls. Among various nutritional elements, salt and fiber have been extensively studied for their role in gut health. Thus, we assessed the amount of dietary sodium (Na^+^) and found that there were no differences between the POTS and HC groups (Figure 1F). The levels of dietary fiber were also similar between groups (Figure 1G).

**FIGURE 2:**
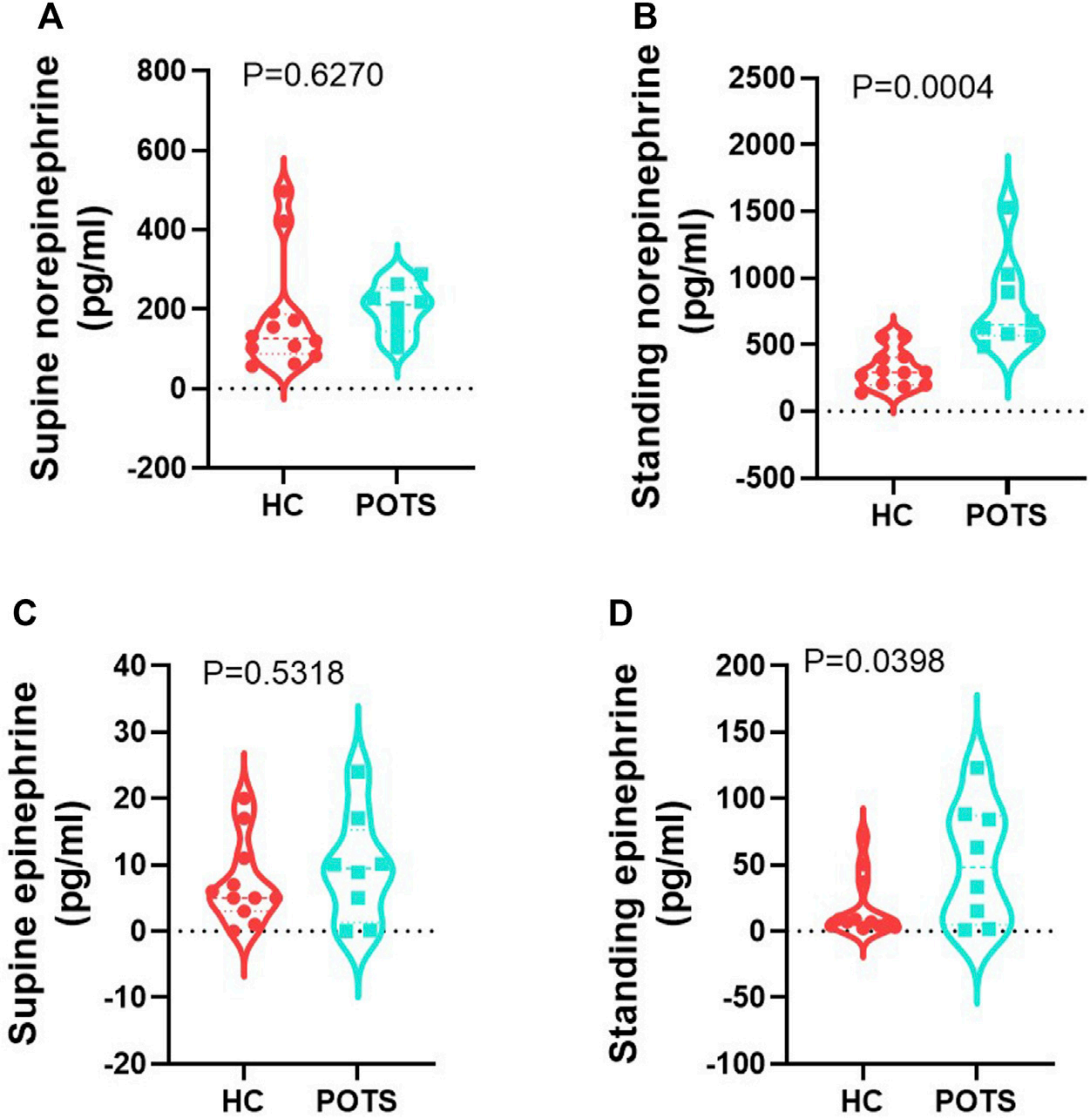
POTS patients have higher catecholamines in the standing position. Circulating norepinephrine in the **(A)** supine and **(B)** standing position and epinephrine in the **(C)** supine and **(D)** standing position. Data are presented as mean ± SEM. Statistical analyses were performed using Student’s *t*-test. *N* = 8 for POTS and *N* = 12 for healthy control participants and *p* < 0.05 was set for statistical significance.

**FIGURE 3:**
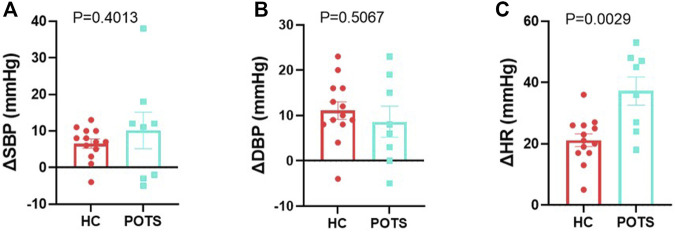
POTS patients undergo orthostatic changes from supine to standing position. Delta change in **(A)** systolic, **(B)** diastolic blood pressure, and **(C)** heart rate measured in POTS and controls between measurements in the standing position and those taken in the supine position. Data are presented as mean ± SEM. Statistical analyses were performed using Student’s *t*-test. *N* = 8 for POTS and *N* = 13 for healthy control participants and *p* < 0.05 was set for statistical significance.

Orthostatic changes: We conducted a posture study to assess disease status in participants by measuring supine and standing systolic and diastolic pressure and heart rate. We assessed orthostatic changes as the difference between parameters measured in the supine position and those measured at the end of the 10 min standing. We also measured catecholamines in blood specimens in the supine and standing positions. Supine norepinephrine (Figure 2A) and epinephrine (Figure 2B) were similar between groups. In the standing position, both catecholamines significantly increased in POTS patients compared to the healthy controls (Figures 2C,D). We measured changes in blood pressure and found that no changes in either systolic (Figure 3A) or diastolic (Figure 3B) pressure occurred between the supine and standing. POTS is associated with a rapid increase in heart rate that occurs within 10 min upon standing. In this study, heart rate significantly increased as evidenced by a high delta change between heart rate measurement taken in the supine position and after 10 min of standing (Figure 3C).

**FIGURE 4:**
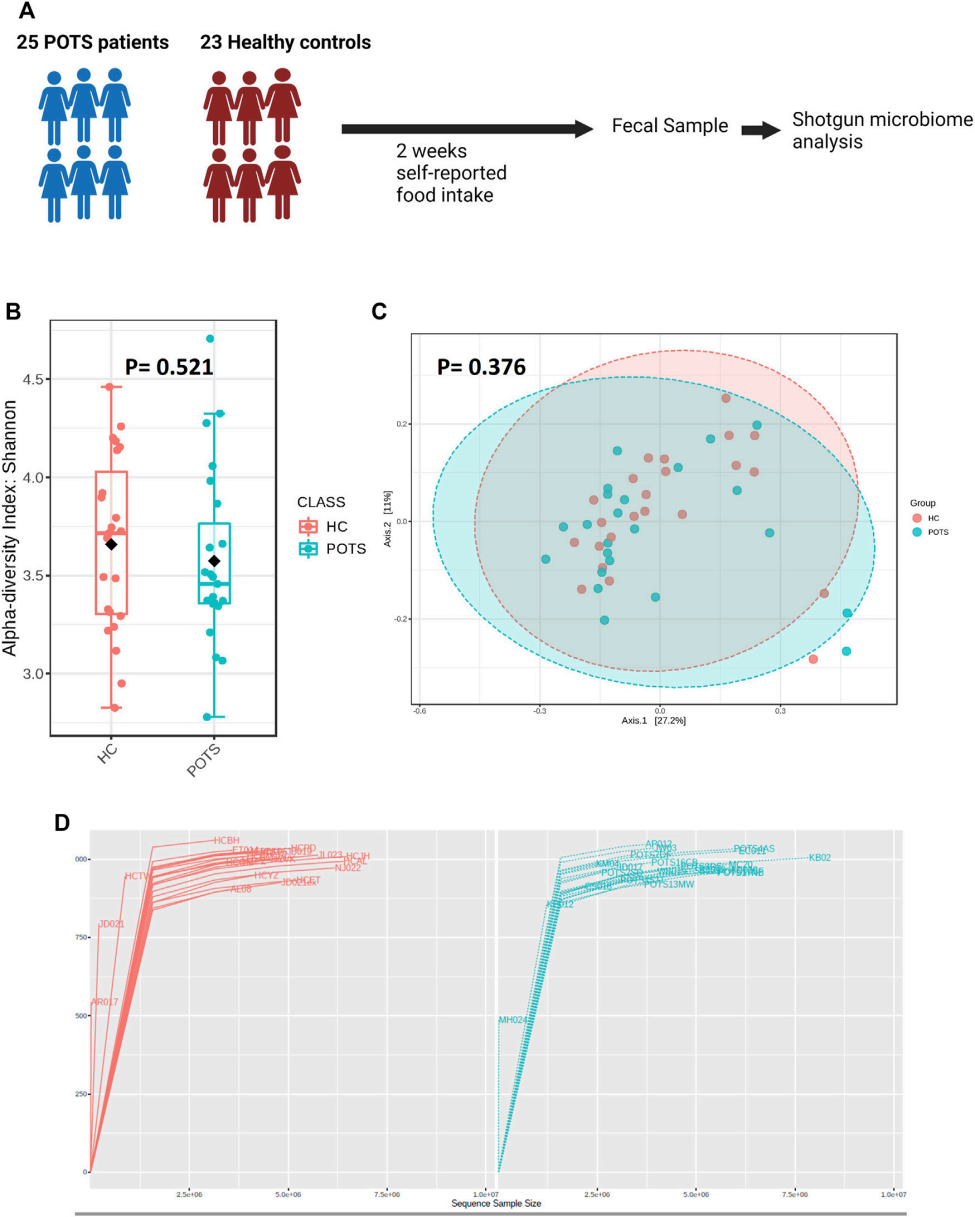
Relationship between POTS and the gut microbiome. **(A)** Study design, the self-reported dietary composition was estimated from data recorded in the MyFitnessPal application for 2 weeks **(B)** Alpha diversity is presented as the Shannon index. **(C)** Principal coordinate axis representing beta diversity measure in POTS patients versus healthy controls **(D)** Rarefaction curve of OTUs in fecal samples. Shotgun sequencing data analyses were performed using MicrobiomeAnalyst and *p* < 0.05 was set for statistical significance. Data are presented as mean ± SEM. Statistical analyses were performed using Student’s *t*-test. *N* = 8 for POTS and *N* = 13 for healthy control participants and *p* < 0.05 was set for statistical significance.

Overall microbiome profiling: Gut microbiota dysbiosis is widely associated with diseases including chronic fatigue syndrome, but its involvement in POTS has not been studied. To determine whether POTS is associated with gut microbiota dysbiosis, we analyzed gut microbiota composition in subjects with POTS and healthy controls using Shotgun sequencing. The study design is shown in Figure 4A. Our analysis showed that Alpha diversity by the Shannon Index, which evaluates the diversity within each sample, was similar (*p* = 0.521) between POTS patients and healthy controls (Figure 4B). Similarities in individual sample diversity are further reflected by similar rarefaction curves between POTS patients and healthy control subjects (Figure 4D). We next assessed beta diversity by Bray-Curtis index using the principal coordinate analysis and observed that the two ellipses overlapped and were similar (*p* = 0.376) between the groups (Figure 4C).

**FIGURE 5:**
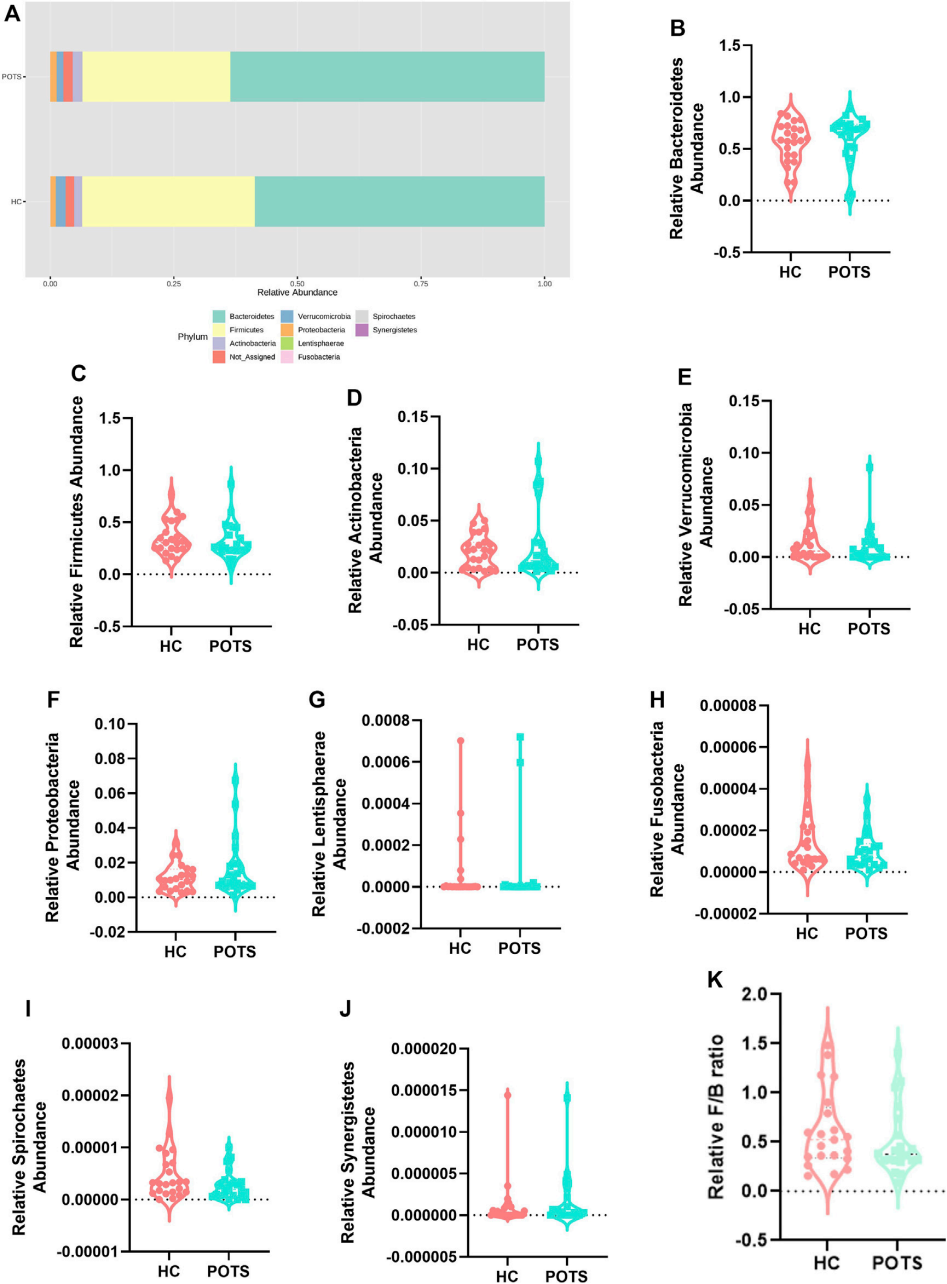
POTS patients do not exhibit dysbiosis at the phylum taxonomic classification. **(A)** Stacked bar plot depicting the relative abundance of phyla in POTS and healthy control participants. Relative abundance of the phyla **(B)** Bacteroidetes; **(C)** Firmicutes; **(D)** Actinobacteria; **(E)** Verrucomicrobia; **(F)** Proteobacteria; **(G)** Lentishphaerae; **(H)** Fusobacteria; **(I)** Spirochaetes; **(J)** Synergistetes; and **(K)** Firmicutes to Bacteroidetes ratio. Data are presented as mean ± SEM. Statistical analyses were performed using Student’s *t*-test. *N* = 25 for POTS and *N* = 23 for healthy control participants and *p* < 0.05 was set for statistical significance.

To determine whether POTS is associated with a distinct microbiota profile, we assessed the relative abundance of bacteria at the phylum and genus taxonomic levels. Overall, we found that the phyla with assigned classification were abundant in the descending order Bacteroidetes; Firmicutes; Actinobacteria; Verrucomicrobia; Proteobacteria; Lentisphaerae; Fusobacteria; Spirochaetes and Synergistetes (Figure 5A). The relative abundance of all investigated phyla was also similar between groups (Figures 4, 5). Previous studies have used the Firmicutes to Bacteroidetes ratio (F/B) as a crude marker for gut dysbiosis. We found no differences in the F/B ratio between POTS and controls (Figure 5K).

**FIGURE 6:**
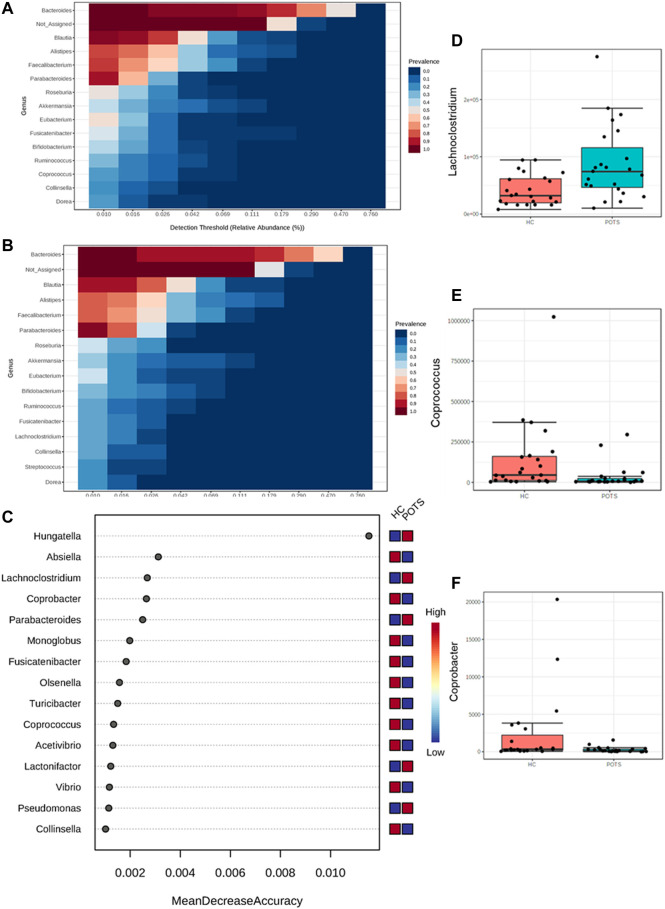
POTS is associated with differences at the genus level in the gut microbiome. The gut microbiome in fecal samples from POTS patients or healthy control subjects was analyzed. **(A)** Heatmap of 15 most abundant genera in healthy controls subjects. **(B)** heatmap of 15 most abundant genera in POTS patients. **(C)** Random forest analysis of POTS and healthy controls genera. Relative abundance of genera **(D)** *Lachnoclostridium* **(E)** *Coprococcus* and**(F)** *Coprobacter*. Data are presented as mean ± SEM. Statistical analyses were performed using Student’s *t*-test. *N* = 25 for POTS and *N* = 23 for healthy control participants and *p* < 0.05 was set for statistical significance.

Next, we evaluated differences in the gut microbiota at the genus level. Specifically, we looked at heatmaps displaying the 15 most abundant genera in the healthy controls (Figure 6A) and compared them to those abundant in POTS patients (Figure 6B). Further, we conducted a random forest analysis to assess differential bacterial signatures which revealed 15 abundant genera (Figure 6C). We observed trends in relative genera abundance between groups. *Lachnoclostridium* (*p* = 0.010)were higher (Figure 6D) whereas *Coprococcus* (*p* = 0.009)and *Coprobacter* (*p* = 0.032) were lower in POTS patients compared to controls (Figures 6E,F). None of the genera were significant after adjusting for multiple comparison (FDR>0.05). To evaluate whether these trends in bacteria abundance were accompanied by differences in microbial metabolic potentials, we conducted a KEGG pathway analysis. We found 36 metabolic pathways that were significantly different between groups. These included those related to energy, fatty acids and SCFAs (butyrate and propionate metabolism) (Supplementary Table S1).

**FIGURE 7:**
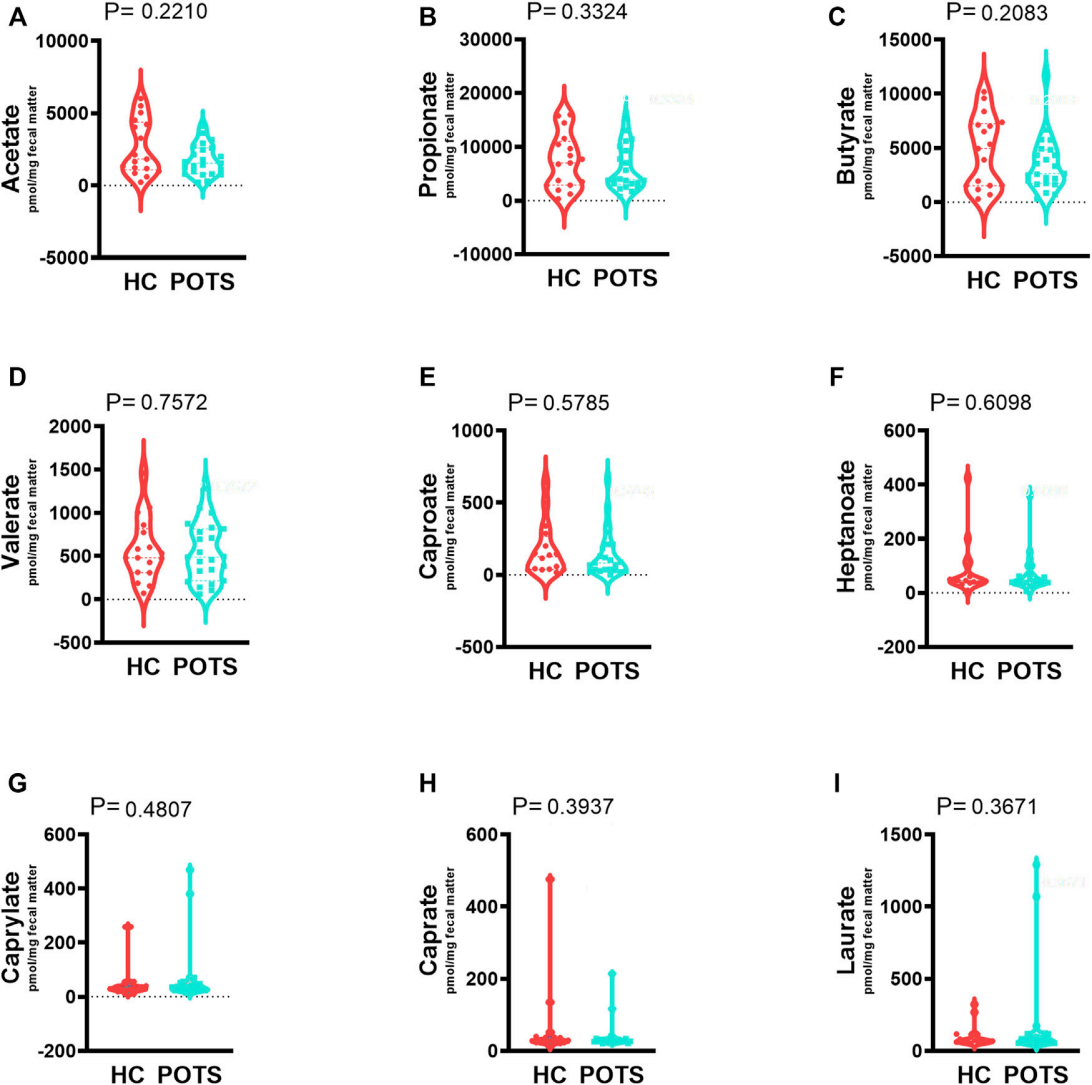
The relationship between POTS and short-chain fatty acids (SCFAs) in the gut microbiome. The fecal samples were collected from control and POTS individuals and the levels of SCFAs were measured with LC-HRMS at Vanderbilt University. We measured fecal levels of the **(A)** acetate; **(B)** propionate; **(C)** butyrate; **(D)** valerate; **(E)** caproate; **(F)** caprylate; **(G)** caprate; **(H)** laurate and **(I)** heptanoate. Our results indicate that POTS patients and healthy controls had similar levels of all measured SCFAs (Figures 7A–H). Data are presented as mean ± SEM. Statistical analyses were performed using Student’s *t*-test. *N* = 25 for POTS and *N* = 23 for healthy control participants and *p* < 0.05 was set for statistical significance.

**FIGURE 8:**
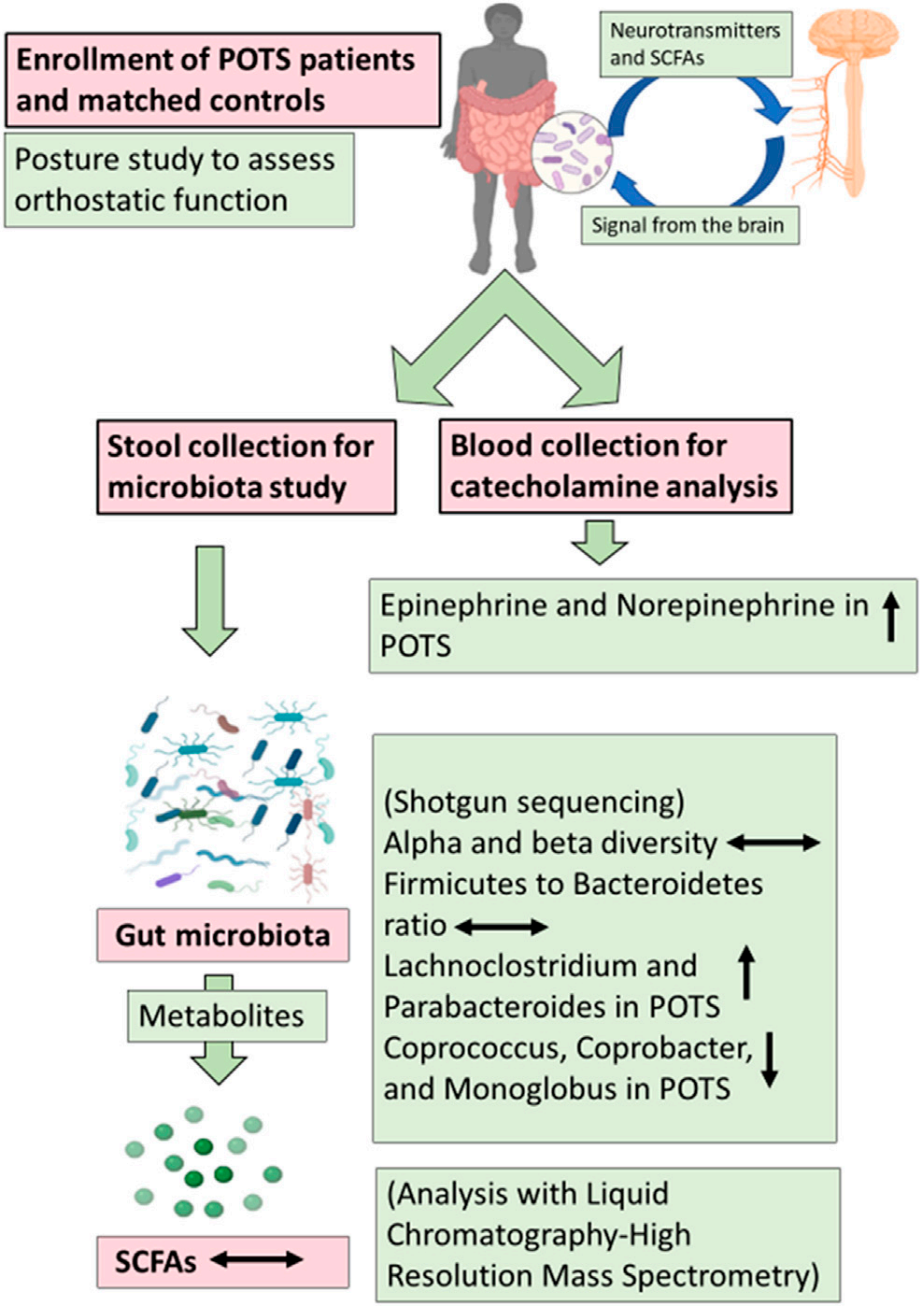
Pictorial depiction of the Summary of the study. POTS patients, 25, and matched controls, 23, were enrolled and a posture study was conducted to assess orthostatic function. Blood and stool samples were collected to perform analysis and gut microbiota was characterized using shotgun sequencing. The metabolites of gut microbiota short-chain fatty acids (SCFAs) were measured with Liquid Chromatography-High Resolution Mass Spectrometry (LC-HRMS).

Short-chain fatty acids analysis: The gut microbiota produces SCFAs which can mediate disease mechanisms. We measured fecal levels of the acetate; propionate; butyrate; valerate; caproate; caprylate; caprate; laurate and heptanoate using LC-HRMS. Our results indicate that POTS patients and healthy controls had similar levels of all measured SCFAs (Figures 7A–H, 8).

## Discussion

POTS is a chronic disabling condition associated with autonomic dysfunction, whose etiology is poorly understood. The autonomic nervous system is intricately connected to the gut and regulates numerous aspects through the enteric nervous system and other gut-brain axis-related mechanisms. While the gut microbiome is now widely acknowledged to play a role in numerous diseases, its role in POTS has not been investigated. In this study, we characterize the gut microbiome profile in POTS. Our results indicate that POTS is associated with increased *Lachnoclostridium* but a reduction in *Coprococcus* and*Coprobacter* compared to healthy controls. Bacteria diversity was similar as were SCFAs. This was a pilot study to investigate the role of the gut microbiome in POTS and our findings provide a rationale for further studies to delineate the specific mechanisms involved.

Diet composition shapes the gut microbiome. In this context, records from 2 weeks of food intake information, total caloric intake, and macronutrients were similar. The gastrointestinal tract health and specific food components such as salt and fiber consumption are closely linked. The American Heart Association recommends ≤2.3 g of sodium per day to avoid the deleterious health effects of excess salt on health [3, 4]. We and others have previously shown that too much salt causes dysbiosis which is associated with inflammation and structural changes in the gut [5, 6]. Unlike the general population, however, subjects who suffer from POTS are recommended to consume 8,000–10,000 mg of dietary salt per day to counteract the hypovolemia (Vernino et al., 2021). A high salt diet decreases heart rate, corrects the hypovolemia, and reduces plasma norepinephrine in POTS patients (Garland et al., 2021). Despite these recommendations, POTS patients consumed similar amounts of sodium as the healthy controls which were below the recommended. Fiber on the other hand promotes a homeostatic microbiota (So et al., 2018; Tanes et al., 2021). We found that fiber intake was also similar between groups. In addition to a high salt diet, POTS patients are recommended to consume increased dietary fiber and complex carbohydrates to reduce blood glucose spikes. Our findings highlight the need to educate POTS patients more about lifestyle changes that can improve their symptoms. Lifestyle changes such as adopting a gluten-free diet, or a high salt diet reduce the symptom burden including gastrointestinal changes in patients with POTS (Smith et al., 2021; Zha et al., 2022).

Our main objective was to evaluate if POTS patients have dysbiosis of the gut microbiota compared with healthy controls. Dysbiosis is reflected in changes in bacterial diversity as well as specific taxonomic changes. The gut microbiota in POTS has not been studied before. Therefore, there are no bacterial changes that are known to be associated with the disease. The overall microbial diversity, both within each individual and between groups, was the same as evidenced by similarities in the alpha- and beta-diversity. POTS patients and healthy controls had similar bacteria profiles at the phylum level as no differences in the relative abundance were noted among phyla. Certain phyla like the Proteobacteria phylum and the ratio between the Firmicutes and Bacteroidetes ratio are commonly associated with various pathologies (Ferguson et al., 2019; Ishimwe et al., 2021) and used to assess dysbiosis. Our study found no differences in the relative Proteobacteria abundance and Firmicutes to Bacteroidetes ratio between groups. Changes in microbiome diversity and dysbiosis indices such as Firmicutes/Bacteroidetes ratio have been reported in other neurological disorders associated with autonomic dysfunction including autism (Osadchiy et al., 2019; Kong et al., 2021).

At the genus level, bacteria in the genera *Lachnoclostridium* were higher in the POTS patients compared to healthy controls. We evaluated whether these trends were associated with metabolic pathway changes and found 37 differentially changed pathways including those related to SCFAs metabolism. Fecal *Lachnoclostridium* has been identified as a potential non-invasive diagnostic marker of colorectal cancer and interventions that improve gut health also attenuate the bacteria (Wang et al., 2018; Liang et al., 2020). On the other hand, genera *Coprococcus and Coprobacter* were decreased in POTS patients. *Coprococcus* is a butyrate-producing bacteria and is associated with increased quality of life in people suffering from depression (Valles-Colomer et al., 2019). *Coprococcus* has anti-inflammatory properties and is also reduced in Parkinson’s disease (Vascellari et al., 2020). Although gut microbiota diversity and phyla were similar, our findings suggest POTS may have a distinct microbial profile at the genus level. More studies are warranted to further characterize the gut microbiota and its role in POTS. Although comparing microbiota findings from different studies can be challenging, we speculate that using tools such as the Strengthening The Organization and Reporting of Microbiome Studies checklist in our future studies will increase the rigor in our results and bridge this gap in the field (Mirzayi et al., 2021).

POTS affects women more than men. It is not known whether the disease affects various races differently. The main limitation is this study is that all the participants were white women. Future studies will further elucidate sex differences in microbiota and SCFAs in a larger and more diverse group of participants. Dietary fiber greatly modulates the gut microbiota and the levels of SCFAs, but the benefits vary among fiber types due to solubility (Marques et al., 2017; Mcrorie and Mckeown 2017; Barber et al., 2020). In this study, we used the MyFitnessPal application to track and collect dietary information. The application generated nutritional summaries. We acknowledge that we were unable to distinguish between soluble, insoluble, and resistant starches. We found no differences in SCFAs between groups suggesting that the forms of consumed fiber were not different between groups either. The effects of fiber on POTS should be examined as this would provide another easily modifiable management modality for these patients.

Dysbiosis of the gut microbiota is associated with a wide range of pathologies, but the mechanisms are still lacking. Evidence points to molecules made and/or modified by bacteria to mediate the physiological effects associated with the gut microbiome. The most studied thus far are SCFAs which are produced by bacteria and released into circulation. Studies in rodents indicate that SCFAs are decreased in depression which positively correlates with changes in neurotransmitters such as norepinephrine (Wu et al., 2020). The findings are also true in depressive women who exhibit lower levels of SCFAs (Skonieczna-Zydecka et al., 2018). Replenishing SCFAs in mice with Alzheimer’s disease leads to structural changes primarily by the microglia cells suggesting that they may play a role in regulating neurological function (Colombo et al., 2021). In this study, we found no differences in the fecal SCFAs acetate; propionate, butyrate, valerate; caproate, caprylate; caprate; laurate, and heptanoate between POTS patients and healthy controls. We speculate that the similarity in fecal levels of SCFAs is a result of the observed strong similarity in the gut microbiome between POTS patients and controls.

This study determined differences in the fecal SCFAs and gut microbiota between POTS patients and healthy controls. Our results supports a conclusion that POTS patients do not have gut dysbiosis or differences in fecal SCFAs. We show an initial characterization of the gut microbiota in POTS and provide a rationale for further well-controlled and diverse studies.

## Data Availability

The datasets presented in this study can be found in online repositories. The name of the repository and accession number can be found below: European Nucleotide Archive (ENA); PRJEB52272.
