# Supplementary material for: The Gut Microbiota and Short-Chain Fatty Acids Profile in Postural Orthostatic Tachycardia Syndrome
Source: Front Physiol. 2022 Jun 2;13:879012. doi: 10.3389/fphys.2022.879012 (PMC9208699; doi:10.3389/fphys.2022.879012)
Supplement: Supplementary file 1 [file Table1.docx]

Pathway	Total	Expected	Hits	Pval	FDR
Biosynthesis of amino acids	223	45.9	104	8.73E-20	1.38E-17
Pyruvate metabolism	86	17.7	44	1.72E-10	1.36E-08
Citrate cycle (TCA cycle)	57	11.7	33	5.32E-10	2.21E-08
Carbon metabolism	299	61.6	105	5.59E-10	2.21E-08
Butanoate metabolism	77	15.9	37	4.60E-08	1.45E-06
Alanine, aspartate and glutamate metabolism	65	13.4	30	2.62E-06	6.90E-05
Propanoate metabolism	82	16.9	35	3.64E-06	8.23E-05
Glycolysis / Gluconeogenesis	82	16.9	34	1.09E-05	0.00022
Arginine and proline metabolism	126	26	46	1.87E-05	0.00033
Pentose and glucuronate interconversions	55	11.3	25	2.53E-05	0.0004
Carbon fixation pathways in prokaryotes	101	20.8	37	0.00012	0.00165
Valine, leucine and isoleucine biosynthesis	15	3.09	10	0.00014	0.00186
Pentose phosphate pathway	67	13.8	27	0.00016	0.00193
Selenocompound metabolism	21	4.33	12	0.00025	0.00279
Starch and sucrose metabolism	87	17.9	32	0.00031	0.00322
Glycine, serine and threonine metabolism	91	18.7	33	0.00033	0.0033
Biotin metabolism	19	3.91	11	0.00039	0.00341
Drug metabolism - other enzymes	19	3.91	11	0.00039	0.00341
Glyoxylate and dicarboxylate metabolism	68	14	26	0.00056	0.00466
Streptomycin biosynthesis	15	3.09	9	0.00096	0.00756
2-Oxocarboxylic acid metabolism	67	13.8	25	0.00108	0.00806
Cysteine and methionine metabolism	78	16.1	28	0.00112	0.00806
Purine metabolism	179	36.9	54	0.00117	0.00806
Synthesis and degradation of ketone bodies	8	1.65	6	0.00143	0.00942
D-Glutamine and D-glutamate metabolism	6	1.24	5	0.00182	0.0108
Geraniol degradation	6	1.24	5	0.00182	0.0108
Fatty acid biosynthesis	28	5.77	13	0.00184	0.0108
Pyrimidine metabolism	85	17.5	29	0.00228	0.0129
Nitrogen metabolism	49	10.1	19	0.00253	0.0138
Carbon fixation in photosynthetic organisms	36	7.42	15	0.00315	0.0166
Nitrotoluene degradation	9	1.85	6	0.00355	0.018
Porphyrin and chlorophyll metabolism	76	15.7	26	0.00364	0.018
Histidine metabolism	38	7.83	15	0.0058	0.0278
Limonene and pinene degradation	5	1.03	4	0.00748	0.0347
Pantothenate and CoA biosynthesis	29	5.97	12	0.00857	0.0387
Glutathione metabolism	40	8.24	15	0.01	0.0439
